# Micron-sized and submicron-sized aerosol deposition in a new *ex vivo* preclinical model

**DOI:** 10.1186/s12931-016-0395-7

**Published:** 2016-07-07

**Authors:** Sophie Perinel, Lara Leclerc, Nathalie Prévôt, Agathe Deville, Michèle Cottier, Marc Durand, Jean-Michel Vergnon, Jérémie Pourchez

**Affiliations:** INSERM, U1059, SAINBIOSE, Saint-Etienne, F-42023 France; Université de Lyon, Saint-Etienne, F-42023 France; CHU Saint-Etienne, Saint-Etienne, F-42055 France; Ecole Nationale Supérieure des Mines de Saint-Etienne, CIS-EMSE, SAINBIOSE, F-42023 Saint Etienne, France; Centre Hospitalier Emile Roux, F-43012 Le Puy en Velay, France

**Keywords:** Aerosoltherapy, Aerosol deposition, SPECT/CT imaging

## Abstract

**Background:**

The knowledge of where particles deposit in the respiratory tract is crucial for understanding the health effects associated with inhaled drug particles.

**Method:**

An *ex vivo* study was conducted to assess regional deposition patterns (thoracic *vs.* extrathoracic) of radioactive polydisperse aerosols with different size ranges [0.15 μm–0.5 μm], [0.25 μm–1 μm] and [1 μm–9 μm]. SPECT/CT analyses were performed complementary in order to assess more precisely the regional deposition of aerosols within the pulmonary tract.

Experiments were set using an original respiratory tract model composed of a human plastinated head connected to an *ex vivo* porcine pulmonary tract. The model was ventilated by passive expansion, simulating pleural depressions. Aerosol was administered during nasal breathing.

**Results:**

Planar scintigraphies allowed to calculate the deposited aerosol fractions for particles in the three size ranges from sub-micron to micron The deposited fractions obtained, for thoracic *vs.* extra-thoracic regions respectively, were 89 ± 4 % *vs.* 11 ± 4 % for [0.15 μm–0.5 μm], 78 ± 5 % *vs.* 22 ± 5 % for [0.25 μm–1 μm] and 35 ± 11 % *vs.*65 ± 11 % for [1 μm–9 μm].

**Conclusion:**

Results obtained with this new *ex vivo* respiratory tract model are in good agreement with the in vivo data obtained in studies with baboons and humans.

## Background

The effectiveness of aerosol drug delivery mainly depends on the amount of active agent penetrating the respiratory tract as well as on its regional distribution. This information is crucial for predicting the beneficial health effects associated with inhaled drug particles. Many inhalation studies were mainly focused on the assessment of the total deposited fraction (often calculated by comparison between inhaled and exhaled fractions) or used computational approaches, for instance, the recent Euler-Lagrangian approach of Kannan et al. [1]. In their study, high fidelity computational simulations were performed over several breathing cycles to get the regional deposition for different particle sizes and an algorithm was devised to account for the re-entry of particles during the exhalation phase. However some attempts were devoted to the quantification of regional particle deposition [2–4]. Nevertheless, in vivo experimental studies on the regional deposition of submicron-sized particles are still quite scarce.

In vivo experiments using radioactive aerosols are widely considered as the gold standard in order to assess the regional deposition of airborne particles. However, obtaining rapidly an extensive experimental dataset in human using radioactive aerosols is difficult and questionable due to ethical restrictions. Thus, in vivo laboratory animal studies are an attractive alternative. On the one hand, rodents, like rats or mice, that are most often used for such inhalation experiments [5, 6] are disparate from the human airways by size, bronchial divisions, anatomy of upper airways and respiratory physiology [7]. As an example, the respiratory rate at rest is around 80 breaths per minute for rats compared to 15 breaths per minute for humans. In these conditions, researchers have to be careful when translating results from in vivo experiments on rodents to humans even if they are helpful to understand deposition mechanisms. On the other hand, radioactive aerosol deposition studies have also been performed using pigs or non-human primates because of their anatomical proximity with human airways [8–11]. However, the main limits of these in vivo laboratory animal studies are ethical restrictions as well as cost and technical constraints (e.g.*,* anesthesia that is often necessary to perform inhalation experiments, specialized laboratories, etc.). Furthermore, the breathing pattern, known to be an important factor for inhalation efficiency (Inspired/Expired ratio, frequency, obstruction etc.), is difficult to control in spontaneously breathing pigs or non-human primates.

As an alternative to in vivo inhalation experiments on humans and laboratory animals, *ex vivo* anatomical models such as cadaver heads [12] or in vitro nasal casts using prototyping techniques [13, 14] have been developed. These models are very accurate to assess aerosol drug deposition in nasal cavities such as maxillary sinuses [15–19]. Nasal casts are morphologically close to the human anatomy and easier to handle compared to in vivo preclinical models. However, even if these replicas are useful for inhalation studies in nasal cavities, very few in vitro or *ex vivo* anatomical models mimic a human-like respiratory tract including both extrathoracic [ET] and thoracic [TH] regions and a physiological breathing pattern.

This article focuses on an original solution to bridge the existing gap on anatomical respiratory models to assess regional aerosol deposition. This study used an *ex vivo* human-like respiratory tract model [20]. This chimeric model is composed of a human plastinated head linked to an *ex vivo* porcine pulmonary tract ventilated artificially by inflation with a negative pressure inside a sealed enclosure (passive expiration). This cheaper and original approach without ethical restriction allows assessing regional aerosol deposition in both extrathoracic and pulmonary regions.

The clinical context of this study is the topical nasal drug delivery. When developing a drug product for nasal delivery, the aerodynamic features of the formulation have to ensure that drug particles are retained in the nasal cavity and not inhaled into the lung. For example, airborne drug delivery to the maxillary sinuses requires variable pressure application (known as ‘pulsating airflow’ aerosols) [15–19, 21] but also small droplets (with a MMAD lower than 5 μm) [22]. The main challenge consists of delivering a high concentration of medication into the maxillary sinus cavities while at the same time preventing unwanted lung deposition observed with small particle delivery systems in order to minimize side effects. Therefore, the optimal droplet size for nasal administration should be based, among other things, on an accurate assessment of aerosol regional deposition in both extra thoracic and pulmonary regions.

The experiments were conducted using radioactive polydisperse aerosols in the [0.15 μm–0.5 μm], [0.25 μm–1 μm] and [1 μm–9 μm] ranges. We used exclusively a nasal administration of aerosol. We measured regional deposition in the TH region versus the ET region using planar scintigraphies and single-photon emission computed tomography (SPECT) [11, 13]. The secondary objective was to evaluate the reliability of this *ex vivo* model for aerosol deposition by comparison with known in vivo data in humans and baboons using the same polydisperse radioactive aerosols and similar nasal administration [11, 17].

## Methods

### Anatomical model

**Fig. 1:**
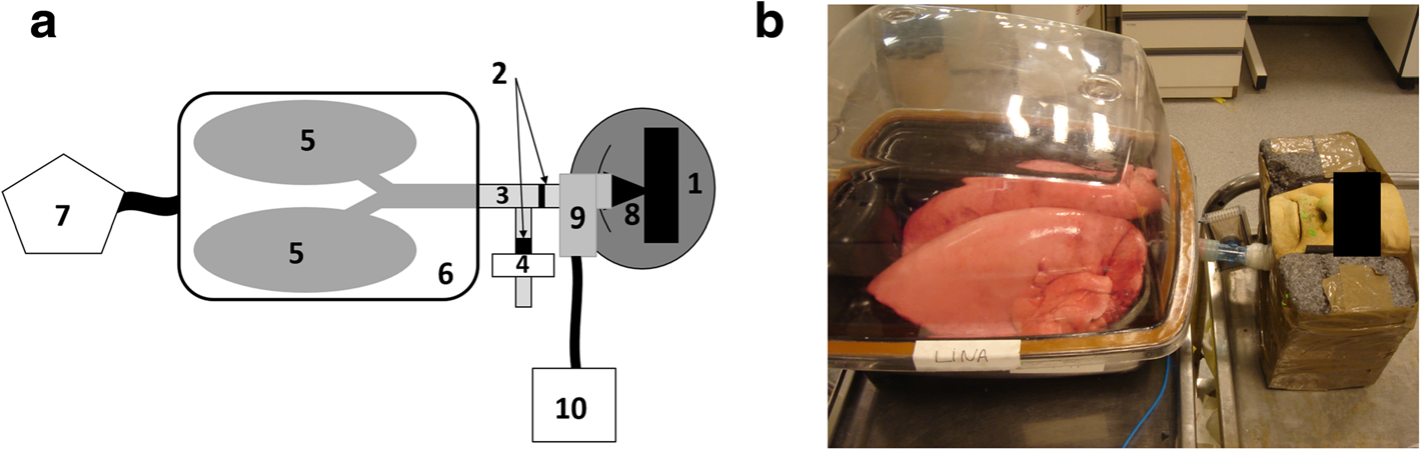
**a** Schematic of the experimental setup for nebulization using the human-like *ex vivo* preclinical model. 1) plastinated human head, 2) one-way valves, 3) plastic tubes, 4) expiratory filter, 5) porcine pulmonary tract, 6) plastic sealed enclosure, 7) respiratory pump, 8) nasal plug, 9) jet nebulizer, 10) compressor. **b** Photograph of the setup before the start of the aerosol experiments

Experiments were performed using an *ex vivo* respiratory tract model. This model is composed of a human plastinated head linked to an *ex vivo* porcine pulmonary tract ventilating artificially by passive expansion with depressions. The laryngeal part is made of plastic tubes with a one-way valve simulating the resistance of vocal folds as inhalation is ongoing. An other one-way valve allows the path for exhalation as previously described [13]. The human plastinated head was anatomically, geometrically and aerodynamically characterized [12]. Physiological parameters obtained were consistent with the widely accepted human physiology [20]. Experimental conditions used were biomimetic with an original breathing technological process simulating the intrapleural depressions. Indeed, the chimeric model respired artificially by inflation with a negative pressure (passive expiration) applied inside an instrumented sealed enclosure developed specifically for this model [20]. The inhalation is due to the depression inside the instrumented sealed enclosure and the exhalation is passive by the return to atmospheric pressure by a pause of the depression generator. Moreover the duration of inhalation and exhalation in the respiratory cycle are chosen with shorter time duration for inhalation. In order to fit the human physiology, a ratio of 1.3 s for inhalation and 2.6 s for exhalation has been chosen, leading to a respiratory cycle of 4 s and consequently a respiratory rate of 15/min. In these conditions, the breathing parameters selected correspond to adult male physiology at rest: breathing rate of 15 breaths per minute, calculated mean tidal volume [VT] of 824 mL (SD 207 mL), inspiratory-to-expiratory time [I/E] ratio of 1/2, ET deadspace of 21 mL, tracheal internal diameter of 1.7 cm [23–25]. The chimeric model satisfied the major functional and anatomical features necessary to study deposition of airborne particles [20]. The experimental setup for inhalation experiments is described in Fig. 1. The position of the exhalation filter is due to a particularity of the plastinated head (closed mouth) so obtained results correspond to an approximation of the exhaled fraction.

### Aerosol generation and characterization

Medical jet nebulizers are used to generate radioactive polydisperse aerosols from sub-micron to micron-sized particles. The nebulizing systems chosen are an Atomisor NL11 (DTF Medical, France), a modified Sidestream (Philips Respironics, Ref 12NEB400, England), and a Nanoneb (DTF Medical, France) operating at a flow rate of 6 L/min. The same experimental conditions concerning the generation of radioactive polydisperse aerosols were used as in previous in vivo studies performed in humans and baboons [11, 17].

Briefly, the nebulizer (Atomisor NL11, Sidestream or Nanoneb) was associated with a same AOHBOX air source compressor (Diffusion Technique Française, DTF Medical, Saint-Etienne, France). The nebulizers were connected to a nasal plug (C28 medium size, DTF Medical, France) usually employed in clinical practice. This plug ensures the interface connection between the nebulizer and plastinated head nostrils. For each experiment, nebulizers (*n* = 4) were loaded with 4 mL of a solution containing 740 MBq of ^99m^Tc-DTPA (TechneScan DTPA, Mallinckrodt Medical, Petten, the Netherlands) and the duration of nebulization was standardized to 10 min as previously described [13, 18, 19, 21].

Radioactive aerosols are well characterized in terms of particle sizing and aerosol output according to protocols developed in previous works [11, 13]. Succinctly, particle size distributions of radioactive aerosols were determined using a gamma camera (Ecam; Siemens, Germany) coupled to a specific electrical low-pressure impactor (ELPI; Dekati, Finland) using the DLPI mode (without the corona charger of ELPI instrument). The DLPI mode allows avoiding possible biases induced by the corona charger (particle size selection phenomena and possible particle losses). The particles were impacted depending on their inertia-related aerodynamic diameter on one of the 12 size fraction stages of the impactor in the 30 nm - 10 μm range. Finally the radioactivity from aerosol particles deposited in each size-specific stage was quantified by scintigraphic imaging. It allowed direct tracing of the activity distribution as a function of aerosol aerodynamic size. Thus, the knowledge of the radioactivity dose deposited on each impactor stage permitted the calculation of:

- the activity median aerodynamic diameter (AMAD) with geometric standard deviation (GSD),
- the total dose of radioactivity delivered by the nebulizer (i.e. the nebulizer emitted fraction),
- the fine particle (FP) fraction (including all particles with an aerodynamic diameter <2.5 μm) and the ultrafine particle (UFP) fraction (including all particles with an aerodynamic diameter <0.1 μm),
- and the [d16–d84] particle size range (where d16 and d84 are respectively the particle diameters at the 16 and 84 % size cut-offs of the cumulative distribution).

### Aerosol regional deposition

#### 2D gamma-imaging acquisition and image analysis procedure

The planar scintigraphic images (matrix 256*256) were recorded with a variable angle dual detector SPECT/ CT (SYMBIA T2; Siemens, Knoxville, TN) equipped with a low-energy, high-resolution collimator (FWHM 8.3 mm at 10 cm); tested weekly for uniformity (UFOV 533 mm × 387 mm, CFOV 400 mm × 290 mm).

**Fig. 2:**
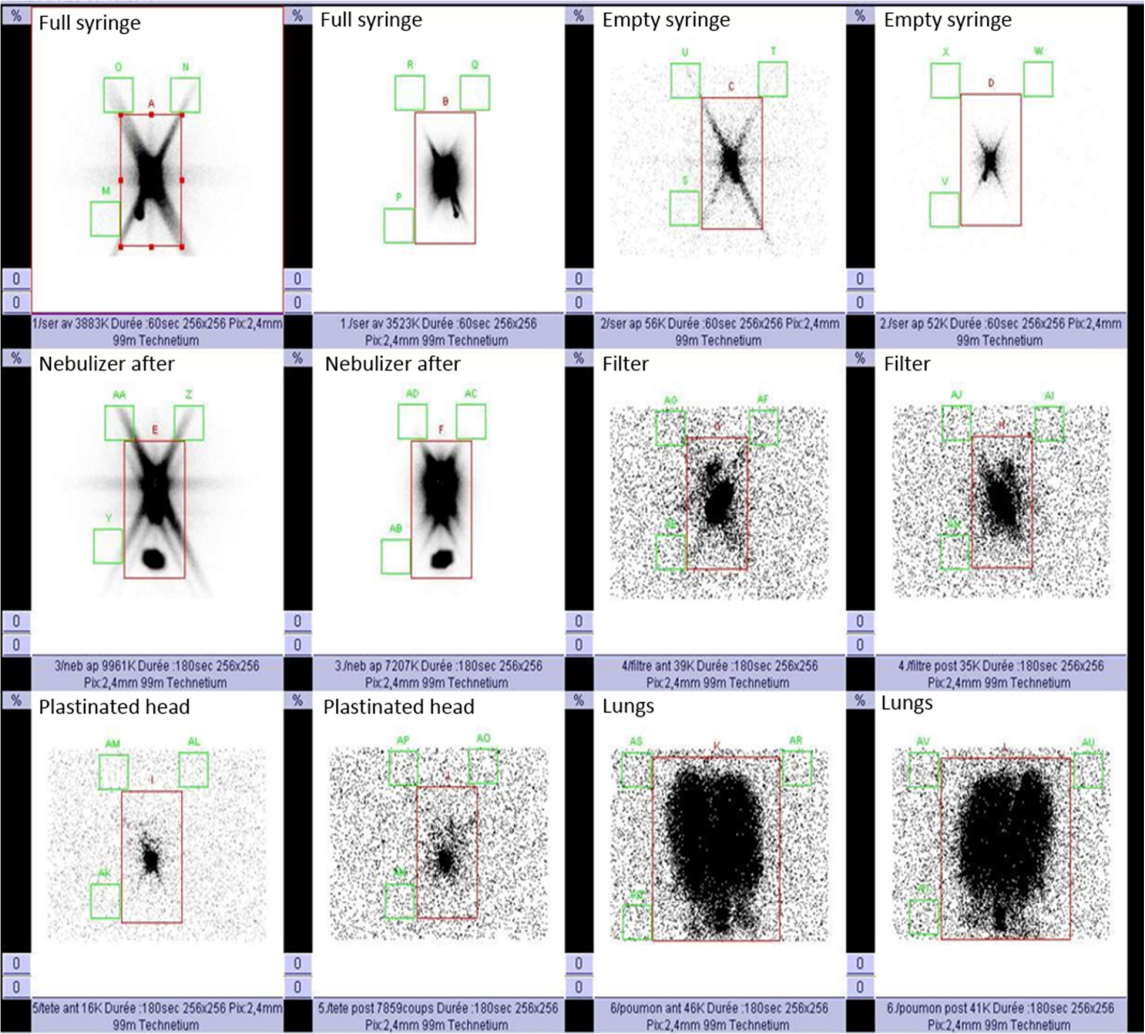
Definition of the 2D imaging anatomical regions of interest (ROIs). Illustrative images of the planar scintigraphies obtained after each experiment. ROIs are delimited in red and the three green squares on each image correspond to the background evaluations

Before conducting the inhalation experiments, the initial radioactive dose filled in the nebulizer was quantified (scintigraphic images, 60-sec anterior/posterior, were acquired corresponding to the full and empty syringe). Once the inhalation experiments were performed, 180-sec anterior/posterior images of the experimental setup were acquired for each element: empty nebulizer, expiratory filter, human plastinated head and lungs as shown on Fig. 2.

A region of interest (ROI) was delimited on the images with a correction of the background using the mean of three external ROIs. Calculations took into account the background radiation, physical decay of radioactivity and tissue attenuation correction factors.

Results were expressed in terms of the activity loaded into the nebulizer and the ratio between TH and ET regions corresponding respectively to the lungs with the tracheobronchial tree and to the head with the artificial larynx.

#### 3D gamma-imaging acquisition and image analysis procedure

After acquiring 2D images with the same gamma camera (SYMBIA T2; Siemens, Knoxville, TN), SPECT and CT acquisitions were performed immediately (without moving the lungs) which have a 2-slice spiral CT for a more rapid and accurate attenuation correction and anatomical mapping. A 3D SPECT acquisition of the lungs was performed with 64 (2 × 32) projection images, each of 30s. Finally, a CT was performed with the following parameters: 130 kV, 90 mAs, 1.25 mm slice thickness, 0.9 mm increment, 1.6 mm pitch, and rotation time of 1.5 s. A multimodality computer platform (Symbia net; Siemens) was used for image reviews and manipulations. Both the transmission and emission scans were reconstructed using 3D OSEM by default (8 subsets, 5 iterations), with pre-reconstruction smoothing using a 3D Butterworth filter (cutoff: 0.45 cycles/cm; order 5), a 128 × 128 image matrix, a 1.23 zoom, and a pixel size of 3.9 mm. SPECT images were reconstructed using scatter correction (scatter energy window) and CT attenuation correction. CT and SPECT images were matched and fused into trans-axial images. A multimodality computer platform (Symbia net; Siemens) was used for image review and manipulation. Different 3D ROIs were drawn on the CT images with the software to delimit peripheral areas from proximal tracheobronchial areas of the lungs.

## Results

### Particle size distribution

As summarized in Table 1, the nebulizing systems led to different AMAD from sub-micron to micron sized particles: 2.8 μm (GSD of 3.2) for the Atomisor NL11, 550 nm (GSD of 2.1) for the Sidestream, and 230 nm (GSD of 1.6) for the Nanoneb. The geometric standard deviation clearly showed that the particle size distributions are broad and quite far from a monodisperse distribution. Thus the aerosol distribution can be characterized by the means of the [d16–d84] particle size range: [1 μm–9 μm] for Atomisor NL11, [0.25 μm–1 μm] for the modified Sidestream and [0.15 μm–0.5 μm] for the Nanoneb.

**Table 1:** Features of the inhaled aerosols to assess the impact of particle size on the regional distribution

Nebulizers	AMAD	GSD	Aerosol output	Particles <1 μm	Particles <0,5 μm	[d16–d84]
NL11	2.80 μm	3.2	44.5 ± 1.5 %	13.6 ± 1.6 %	4.7 ± 0.6 %	[1 μm–9 μm]
Sidestream	550 nm	2.1	9.3 ± 3 %	89.2 ± 2 %	47.9 ± 4.4 %	[0.25 μm–1 μm]
Nanoneb	230 nm	1.6	4.2 ± 1 %	97.5 ± 0.5 %	86.6 ± 1.8 %	[0.15 μm–0.5 μm]

### Regional aerosol deposition by 2D gamma-imaging

**Fig. 3:**
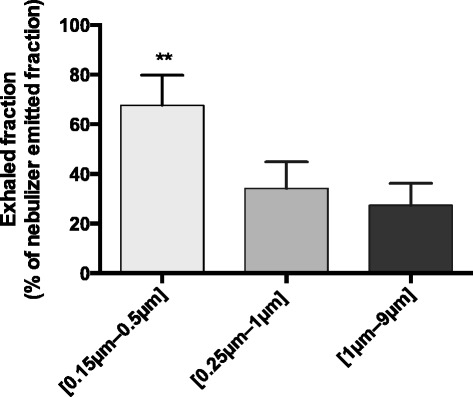
Respective percentages of exhaled fractions obtained for each [d16–d84] size range: [0.15 μm–0.5 μm], [0.25 μm–1 μm] and [1 μm–9 μm]. Results are expressed in ratio (%) of the nebulizer emitted fraction (mean ± SD, **p < 0,01)

The ROIs determined by 2D imaging after planar scintigraphies allowed to quantify the ET and TH deposited fractions (Table 2) but also an approximation of the exhaled fraction (Fig. 3) after the nasal administration of the polydisperse aerosol.

**Table 2:** Deposited fractions in extrathoracic (ET) and thoracic (TH) regions for the different types of polydisperse radioactive aerosols. Results are expressed in ratio (%) of the total deposited fraction (mean ± SD). Comparison of experimental results obtained with the chimeric model to baboon and human in vivo experimentations realized in previous studies [11, 17]

		[0.15 μm–0.5 μm]	[0.25 μm–1 μm]	[1 μm–9 μm]
Chimeric model (Supine position)	ET	11 ± 4 %	22 ± 5 %	65 ± 11 %
	TH	89 ± 4 %	78 ± 5 %	35 ± 11 %
Human (Erect position)	ET	NC	NC	72 ± 14 %
	TH	NC	NC	28 ± 12 %
Baboon (Erect position)	ET	16 ± 4 %	49 ± 8 %	72 ± 17 %
	TH	84 ± 4 %	51 ± 8 %	28 ± 17 %

Besides, the exhaled fraction decreased as the size of aerosol increased. More precisely, for the [0.15 μm–0.5 μm] aerosol particles, we noticed a very significant exhaled fraction (68 ± 12 %, expressed as a percentage of the emitted aerosol fraction by the nebulizer). In contrast, the exhaled fraction for the [0.25 μm–1 μm] and the [1 μm–9 μm] aerosol particles was quite similar (respectively 34 ± 11 % and 27 ± 9 %).

ET deposited fractions obtained for the three types of polydisperse aerosols were 11 ± 4 % for the [0.15 μm–0.5 μm] size range, 22 ± 5 % for [0.25 μm–1 μm] range and 65 ± 11 % for [1 μm–9 μm] range. In the same way, TH deposited fractions were 89 ± 4 % for the [0.15 μm–0.5 μm] size range, 78 ± 5 % for [0.25 μm–1 μm] aerosol and 35 ± 11 % for [1 μm–9 μm] aerosol. These data were compared to known in vivo data obtained in previous studies with baboons and humans (Table 2) using similar experimental conditions [11, 17].

### Pulmonary deposition assessed by SPECT/CT

**Fig. 4:**
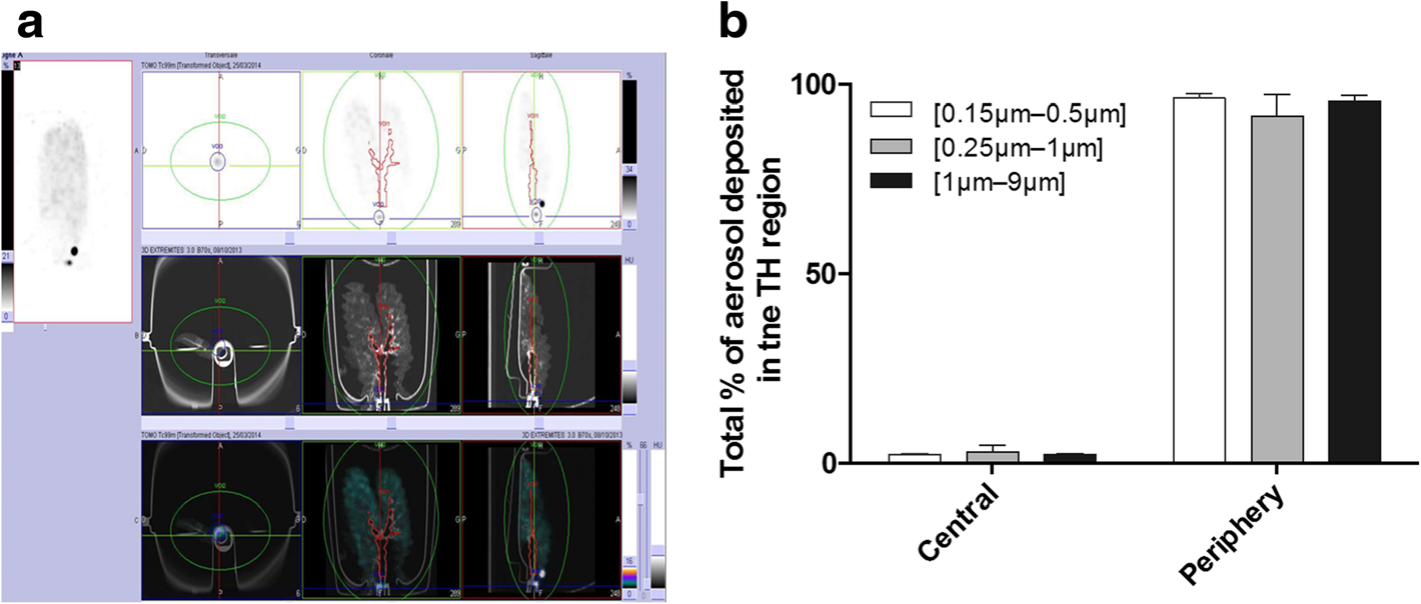
**a** SPECT/CT imaging of the lung and delimitation of the central and peripheral regions of interest (*red*). **b** Percentage of deposition in the central and peripheral regions obtained for each [d16–d84] size range: [0.15 μm–0.5 μm], [0.25 μm–1 μm] and [1 μm–9 μm]

The 3D anatomical ROIs determined by CT scans with radioactivity counted by SPECT are shown in Fig. 4. With this methodology, the ROI outlines allowed the delimitation of the proximal and peripheral regions of the lungs. The proximal region corresponds to the trachea and the two principal bronchi. Representative SPECT-CT images of regional lung deposition obtained for the different polydisperse aerosols are shown in Fig. 4a. Interestingly, there were no significant differences among the three types of polydisperse radioactive aerosols [26]. Indeed, a large deposition of aerosols was clearly visible in the peripheral lung region whereas a low amount of radiolabeled airborne particles was deposited in the proximal tracheobronchial region (less than 5 % of the total aerosol deposition). The percentages of aerosol deposited in the lung (see ET fraction in Table 2) were normalized to 100 % to compare the proximal (central) versus peripheral regions of the lungs and are illustrated in Fig. 4b. The thoracic deposition percentage varies significantly in the model depending on the AMAD but whatever the thoracic deposition amount, this deposit is still peripheral.

## Discussion

In this study, the use of the chimeric model appeared useful in performing regional aerosol deposition cartographies for nasally administered aerosols. Moreover, the ET and TH deposited fractions for particles in the [1 μm–9 μm] and the [0.15 μm–0.5 μm] range were in acceptable agreement between inhalation experiments on baboons, healthy human volunteers and the chimeric model (i.e. respectively 72 ± 17 %, 72 ± 14 %, 65 ± 11 % for ET deposited fraction for the [1 μm–9 μm] size range) with non significant statistical differences (*p* = 0.4799). By contrast, significant differences (*p* < 0.0001) were noticed for the [0.25 μm–1 μm] aerosol (i.e. 22 ± 5 % in the chimeric model versus 49 ± 8 % in baboons for the ET deposited fraction).

Anatomically, the respiratory tracts of pigs are very similar to those of humans, with the same 23 branching divisions except for position of the tracheal bronchus, making pigs very good animal models of the human respiratory tract for decades [27]. Because its half-life after death is a few hours, surfactant is missing in the *ex vivo* porcine lung. This had to be taken into account in our experimental conditions. The simulated intra-pleural depressions necessary to inflate the lungs in the sealed enclosure were adjusted to compensate the absence of surfactant. As a result, the model requires twice the values of depression usually obtained for intrapleural depression during human breathing, which is consistent with the biomechanical role of the surfactant (approximately 50 % of the compliance) [28]. It has to be noted that the absence of blood circulation in this model impacts the compliance too.

Besides, limitations of inhalation experiments using the chimeric model have to be underlined which could explain differences observed for airborne particles in the [0.25 μm–1 μm] range.

On the one hand, the flap valve installed to simulate the vocal folds may filter larger particles much less or more effectively than real vocal folds depending on the occluded area and the flexibility. Nevertheless, the valve’s size and resistivity were carefully selected and this system has already been described and used in previous studies [13, 18]. Moreover previous studies using the proposed model were for gas ventilation (radioactive krypton) experiments without aerosols (i.e. particles or droplets suspended in the air). These points have to be taken into account for the extrathoracic/thoracic results reported in Table 2.

On the other hand, in the chimeric model, the inhalation mode and postural conditions vary from the compared studies (in vivo inhalation experiments with baboon and healthy human volunteer):

- supine position with a nasal administration of the aerosol was used during the experiments with the chimeric model.
- erect position with a nasal administration of the aerosol for the in vivo human experiments.
- erect position with an oro/nasal administration for the in vivo baboon experiments.

For a given airborne particle size range and thus a specific deposition mechanism, this variation of inhalation mode and postural condition can play an important role and have a direct impact on comparisons between baboon, human and the chimeric model. Indeed, airborne particles can deposit by different ways in the respiratory tract, and it is possible to correlate the aerodynamic diameters to the deposition mechanisms in the respiratory airways. Inertial impaction, gravitational sedimentation, and turbulence-driven diffusion mainly govern the deposition of aerosol particles. Each deposition mechanism is directly related to aerodynamic particle size, airway geometry and particle velocity [29–32]. For example, the respiratory deposition of particles in the [1 μm–9 μm], [0.15 μm–0.5 μm] and [0.25 μm–1 μm] size ranges are governed mainly by impaction, turbulence-driven diffusion and sedimentation mechanisms, respectively. Turbulence-driven diffusion mechanism is by random Brownian diffusion mainly in the pulmonary region. Some researchers have recently differentiated a turbulent diffusion mechanism as an addition to their impaction models in the upper airways [33].

It seems interesting to notice that the correlation of in vivo experiments performed in baboon and human compared to the *ex vivo* model is in good agreement for all airborne particle sizes, except those that have a mechanism of deposition by sedimentation mainly due to by gravity, i.e. the [0.25 μm–1 μm] range. Concerning the [1 μm–9 μm] aerosols, the deposition pattern observed in human, baboon and *ex vivo* model is very similar (around 70 % of deposition in the ENT region, *p* = 0,4799, no significant difference). These results confirm that there is no impact of the inhalation mode and the postural conditions when the deposition of aerosols is mainly governed by impaction. Similarly for the [0.15 μm–0.5 μm] aerosols, deposition is comparable between baboon and the *ex vivo* model (around 85 % of deposition in the thoracic region, *p* = 0,0623, no significant difference). These results confirm that there is a minimal impact of the inhalation mode and the postural conditions when the deposition of aerosol is mainly governed by diffusion. In contrast, disparate deposition results were observed for the [0.25 μm–1 μm] aerosols between the chimeric model and studies on baboon (*p* < 0,0001, significant difference). This result suggests that there is a high impact of the respiratory mode and the postural conditions when the deposition of aerosol is mainly governed by sedimentation.

To sum up, for sedimentation mechanism, the postural position (erect or supine) has a significant influence on the regional deposition whereas it is less important for the impaction and diffusion processes where the gravity does not play a major role [34–37]. We assume that the regional aerosol deposition differences observed between the proposed *ex vivo* model and the in vivo experiments for the [0.25 μm–1 μm] aerosols can be mainly attributed to the influence of postural conditions. The limit of the *ex vivo* respiratory tract is the supine position during the inhalation experiment modifying the effects of gravity during the aerosolization compared to in vivo data obtained in the erect position. Thus, the chimeric model appears as quite reliable for predicting regional aerosol deposition for all aerosol size ranges, except for aerosol deposition governed by sedimentation as an inhalation experiment in erect position is needed. Technical solutions to perform the same experiments in erect position are under active consideration progress. The proposed *ex vivo* respiratory tract is an important tool to address the three R’s approach for aerosol deposition studies (Replacement: methods that do not employ animals; Reduction: methods that result in the use of fewer animals than existing methods; Refinement: methods or techniques that reduce pain, distress or discomfort to the animal), encouraging alternatives to in vivo animal testing.

## Conclusion

This study presents original regional deposition data for submicronic and micronic particles in an *ex vivo* respiratory tract and compares these to in vivo data. Results clearly demonstrate a quite good agreement between the regional aerosol deposition obtained for [1 μm–9 μm] and [0.15 μm–0.5 μm] polydisperse aerosols compared to in vivo inhalation experiments in baboons, healthy human volunteers and the *ex vivo* model. However, a bias induced by the use of the *ex vivo* respiratory tract (with experiments currently performed in supine position, see Fig. 1) was noticed for the aerosols with a deposition mechanism governed by gravity (0.25 μm–1 μm range) in comparison with in vivo inhalation experiments performed in the erect position (0.25 μm–1 μm range). In conclusion, this study shows that the *ex vivo* respiratory tract proposed appears as quite reliable to assess aerosol deposition for a wide range of polydisperse aerosols (from sub-micron to micron sized particles), inhalation mode (nasal and oro/nasal administration) and postural positions (supine and erect positions). Thus, the methodology developed is useful to better understand the regional deposition of aerosols within the respiratory tract raising interesting ethical and cost issues, mainly to involve alternative methods as defined by the three R’s approach.

## Abbreviations

3D OSEM, three-dimensional ordered subsets expectation-maximization; ^99m^Tc-DTPA, Technetium 99 m (metastable) Diethylene Triamine Pentaacetic Acid; AMAD, activity median aerodynamic diameter; ELPI, electrical low-pressure impactor; ET, extrathoracic; GSD, geometric standard deviation; I/E ratio, inspiratory-to-expiratory time ratio; MBq, MegaBecquerel; ROI, region of interest; SD, standard deviation; SPECT, single-photon emission computed tomography; TH, thoracic; VT, tidal volume
